# Dysregulated anti-viral innate immune cascade during aging

**DOI:** 10.18632/aging.202588

**Published:** 2021-01-27

**Authors:** Colby Stotesbury, Luis J. Sigal

**Affiliations:** 1 Department of Microbiology and Immunology, Thomas Jefferson University, Philadelphia, PA 19107, USA

**Keywords:** viral infection, aging, innate immunity, dendritic cells, natural killer cells, inflammatory monocytes

Aging predisposes to increased morbidity and lethality to infectious diseases, which becomes apparent with the high mortality rates suffered by older people when infected with influenza virus or severe acute respiratory syndrome coronavirus 2 (SARS-CoV-2). At the root of this increased susceptibility to infections, is the widespread deterioration of the immune system.

Early studies on immunological aging have mostly focused on adaptive immunity and found deficiencies in adaptive lymphocyte development and function. These include decreased T and B cell numbers and reduced effector and recall responses, leading to diminished anti-viral immunity [1]. More recent studies, however, indicate that aging also affects the development and function of innate immune cells in addition to adaptive immunity.

Because the innate immune system plays essential roles in the initial control of pathogens and also in the activation of adaptive immunity, age-associated changes in innate immune cells can potentially have a significant role in the increased susceptibility of the elderly to viral morbidity and lethality. Aging is known to affect human innate immune cells. For example, one study found that dendritic cells (DCs) from aged people infected *in vitro* with West Nile virus had diminished nuclear translocation of STAT1, IRF7, and IRF1, resulting in decreased production of type one interferon (IFN-I) when compared to DCs from young controls [2]. Similarly, monocytes (MO) from aged individuals produced less IFN-I in response to influenza virus compared to MO from young controls [2]. These examples of altered human innate immune cell function suggest impaired anti-viral responses, but due to lack of *in vivo* data, it is challenging to ascertain a causal relationship. However, the use of experimental mouse models provides a useful tool to investigate immunological aging.

Like humans, aging increases the susceptibility of mice to morbidity and death after viral infections. For example, our laboratory has shown that while C57BL/6 (B6) mice survive footpad infection with ectromelia virus (ECTV) without significant disease signs when young, they gradually lose this resistance and all succumb to the infection once they reach 14 months of age. Years ago, we found that a reason for this increased susceptibility is that fewer natural killer (NK) cells get recruited to the popliteal draining lymph node (dLN) of aged mice. The result is a much more rapid systemic viral dissemination and uncontrolled viral replication in the liver and spleen. We also showed that NK cells in aged mice have an immature phenotype compared to young mice. Yet, the adoptive transfer of NK cells from young into older mice only partially restored their survival to ECTV infection [3]. These results indicated that in addition to intrinsic NK cell defects, there might be other deficiencies contributing to the loss of resistance of aged mice to ECTV.

The recruitment of NK cells to the dLN is one of many events that confer young B6 mice with resistance to footpad infection with ECTV. In a series of papers, we identified an intricate network of innate immune responses in the dLN that are indispensable for their resistance. We showed that following ECTV infection of young B6 mice in the footpad, infected and uninfected migratory DCs (mDCs) from the skin migrate to the dLN within the first 16 hours post-infection (hpi). At 24 hpi, infected mDCs in the dLN upregulate NKG2D ligands to activate group 1 innate lymphoid cells (G1-ILC) to produce interferon-gamma (IFN-γ). Simultaneously, infected and uninfected mDCs upregulate expression of mRNA for the chemokines CCL2 and CCL7, which recruit inflammatory MO (iMO) to the dLN. At the peak of iMOs recruitment (60 hpi), iMOs perform distinctive roles depending on their infection status. Uninfected iMOs produce CXCL9 in response to the IFN-γ secreted by the G1-ILCs to recruit NK cells to the dLN. Infected iMOs produce significantly less CXCL9, but are potent producers of IFN-I. Disruption of these events results in increased viral replication and death of infected mice [4–6].

Because of their importance of mDCs and iMOs in recruiting NK cells and producing IFNs, we recently investigated whether aging may alter their function [7]. We found that ECTV fails to induce mDC accumulation in the dLN of aged mice at 24 and 48 hpi. The absence of mDC accumulation results in decreased expression of CCL2 and CCL7 mRNA, resulting in reduced overall recruitment of iMOs to the dLN. Moreover, the lack of mDC accumulation also results in reduced IFN-γ production by G1-ILCs and, as a consequence, increased viral replication in the dLN, higher frequency of infected iMOs, and a drastic reduction in the number of uninfected iMOs that produce CXCL9. The decrease in CXCL9-producing iMOs is likely a contributing factor to the reduced recruitment of NK cells to the dLN [3].

In summary, the lack of mDC accumulation in the dLN during the early stages of ECTV infection in aged B6 mice results in a highly dysregulate innate immune cascade. Consequences are reduced NK cell recruitment, inability to control early virus loads, rapid viral spread, and death from mousepox. Our data highlight previously unknown defects in innate immunity that contribute to increased susceptibility of the aged to viral infection. Future work could determine whether restoring mDC accumulation in the dLN can increase NK cell recruitment and reinstate ECTV resistance to aged mice. The results should open avenues to therapeutic intervention to reinvigorate the immune response in older people and promote their ability to control pathogens more effectively.
